# Internal Herniation Through the Falciform Ligament of the Liver: A Systematic Review of Diagnosis and Operative Strategies

**DOI:** 10.7759/cureus.104343

**Published:** 2026-02-26

**Authors:** Andrew Kelly, Elena Pilat, Connor Augustson, Zac Horstman

**Affiliations:** 1 General Surgery, Cairns Hospital, Cairns, AUS

**Keywords:** computed tomography (ct), emergency general surgery, falciform ligament hernia, internal abdominal hernia, laparoscopic emergency surgery

## Abstract

Internal herniation through a defect in the falciform ligament of the liver is an exceptionally rare occurrence with a risk of small-bowel obstruction that can result in strangulation and bowel necrosis if diagnosis is delayed. Because of its infrequency, available evidence is limited to isolated case reports and small descriptive studies, and optimal diagnostic and operative strategies remain poorly defined.

A systematic search of PubMed/MEDLINE, Embase and Cochrane Library was performed to identify English-language articles published over the past 10 years reporting cases of internal herniation through the falciform ligament. Titles and abstracts were screened using predefined inclusion and exclusion criteria, followed by full-text review. Data were extracted regarding patient demographics, presentation, imaging findings, operative management and outcomes. A qualitative synthesis was undertaken in accordance with Preferred Reporting Items for Systematic Reviews and Meta-Analyses (PRISMA) guidelines.

Seven studies met the inclusion criteria, all of which were single-patient case reports or imaging-focused descriptions. Patients typically presentations varied but included acute abdominal pain and symptoms of small-bowel obstruction, and both congenital and iatrogenic falciform ligament defects were implicated. Computed tomography frequently demonstrated closed-loop bowel positioned anterior to the liver, beak-like tapering at the site of constriction and abnormal displacement of the round ligament. All patients underwent urgent surgical intervention. Management consisted of reduction of the herniated viscus with division or closure of the falciform ligament defect, with bowel resection required when ischemia was present. Both open and laparoscopic approaches were reported, and short-term postoperative outcomes were generally favourable.

Internal herniation through the falciform ligament is a rare but potentially serious surgical emergency that should be considered in cases of unexplained proximal small-bowel obstruction. Recognition of characteristic computed tomography findings may facilitate earlier diagnosis and intervention. Prompt operative exploration remains the cornerstone of management, with ligament division or closure and selective bowel resection based on intraoperative viability.

## Introduction and background

Internal hernias are an uncommon cause of small-bowel obstruction that carries a substantial risk of strangulation, ischemia and mortality when diagnosis is delayed [1,2]. Among the various anatomical subtypes, herniation through a defect in the falciform ligament of the liver is exceptionally rare [3]. The falciform ligament is a peritoneal fold connecting the anterior abdominal wall to the liver and containing the round ligament; defects within this structure may be congenital or acquired, particularly following laparoscopic instrumentation [4,5]. When the bowel becomes entrapped through such an aperture, patients frequently present with nonspecific abdominal symptoms and preoperative diagnosis remains challenging [6]. 

Because of its rarity, falciform ligament internal herniation is reported almost exclusively in isolated case reports and small case series [3,6]. Presentations range from intermittent abdominal pain to acute closed-loop obstruction with bowel necrosis requiring emergent resection [4,7]. Advances in cross-sectional imaging, particularly computed tomography (CT), have led to increasing recognition of characteristic radiologic features, yet most cases continue to be diagnosed intra-operatively [6,8]. Operative strategies also vary, including reduction alone, division or closure of the falciform ligament and bowel resection when ischemia is present, with both open and minimally invasive approaches described [4,6]. 

To date, the literature describing this entity remains fragmented, and no consensus exists regarding optimal diagnostic pathways or surgical management. A contemporary synthesis of reported cases is therefore valuable for practising general and emergency surgeons, particularly given the increasing use of laparoscopy and the potential for iatrogenic falciform ligament defects [5,6].

The aim of this systematic review is to identify and analyse published cases of internal herniation through the falciform ligament reported over the last decade, summarising patient characteristics, clinical presentation, imaging findings, operative management and outcomes. By collating the available evidence, we seek to clarify patterns of presentation, highlight diagnostic clues and inform surgical decision-making for this rare but potentially life-threatening condition.

## Review

Methods 

Protocol and Reporting Standards

This systematic review was conducted in accordance with the Preferred Reporting Items for Systematic Reviews and Meta-Analyses (PRISMA) 2020 guidelines. A predefined review protocol specifying eligibility criteria, search strategy and outcomes of interest was developed prior to the literature search. 

Eligibility Criteria

Studies were considered eligible if they met the following inclusion criteria: published in English; published within the last 10 years; indexed in PubMed/MEDLINE, Embase or Cochrane Library; reported cases of internal herniation through a defect in the falciform ligament of the liver, confirmed radiologically and/or intraoperatively; or study designs including case reports, case series or observational studies. Studies were excluded if they described diaphragmatic hernias (including Morgagni, Larrey or Bochdalek hernias) without true herniation through the falciform ligament; reported ventral, incisional or epigastric hernias containing the falciform ligament rather than internal herniation or focused on surgical use of the falciform ligament as a flap, graft or buttress; involved animal models or cadaveric/anatomical studies without clinical cases; or addressed other forms of internal hernia unrelated to the falciform ligament.

Information Sources and Search Strategy

A systematic search of PubMed/MEDLINE, Cochrane Library and Embase was performed to identify relevant studies published in the preceding 10 years. The search strategy combined controlled vocabulary and free-text keywords relating to the falciform ligament and internal herniation. 

The following search string was used for PubMed/MEDLINE: (“falciform ligament”[Title/Abstract] OR “falciform”[Title/Abstract]) AND (hernia*[Title/Abstract] OR herniation[Title/Abstract] OR “internal hernia”[Title/Abstract] OR obstruction[Title/Abstract] OR strangulat*[Title/Abstract]); for the Cochrane Library: (“falciform ligament” OR falciform) AND (hernia* OR herniation OR “internal hernia” OR obstruction OR strangulat*); and for Embase: (falciform NEXT/1 ligament):ti,ab AND (hernia* OR herniation OR “internal hernia” OR obstruction OR strangulat*):ti,ab.

Search results were filtered by publication date and language. Reference lists of included articles were manually screened to identify additional relevant reports that may not have been captured in the primary search. 

Study Selection

All records retrieved from the database search were exported into a citation manager, and duplicate entries were removed. Two reviewers independently screened titles and abstracts for eligibility. Articles that appeared potentially relevant were retrieved in full text for further assessment. Full-text screening was performed independently by the same reviewers using the predefined inclusion and exclusion criteria. Any disagreements were resolved through discussion and consensus. The study selection process was documented using a PRISMA 2020 flow diagram. 

Data Extraction

Data were independently extracted from included studies by two reviewers using a standardised data-collection form. Extracted variables included the following: first author and year of publication; country of origin; study design; patient age and sex; relevant surgical history; herniated contents; clinical presentation; imaging findings and preoperative diagnosis; operative approach (open, laparoscopic or robotic); surgical procedure performed; requirement for bowel resection; management of the falciform ligament defect; and postoperative outcomes and complications. 

Quality Assessment

Given that most eligible studies were expected to be case reports or small case series, methodological quality and reporting completeness were assessed using appropriate critical appraisal tools for descriptive studies, such as the CARE checklist or the Joanna Briggs Institute critical appraisal instruments. Formal risk-of-bias scoring and quantitative synthesis were not planned because of heterogeneity in reporting and study design. 

Data Synthesis

Due to the rarity of the condition and the predominance of descriptive studies, meta-analysis was not feasible. A qualitative narrative synthesis was therefore performed. Where possible, descriptive statistics were calculated to summarise patient demographics, presentation patterns, imaging characteristics, operative strategies and outcomes. 

Results 

Study Selection

**Figure 1:**
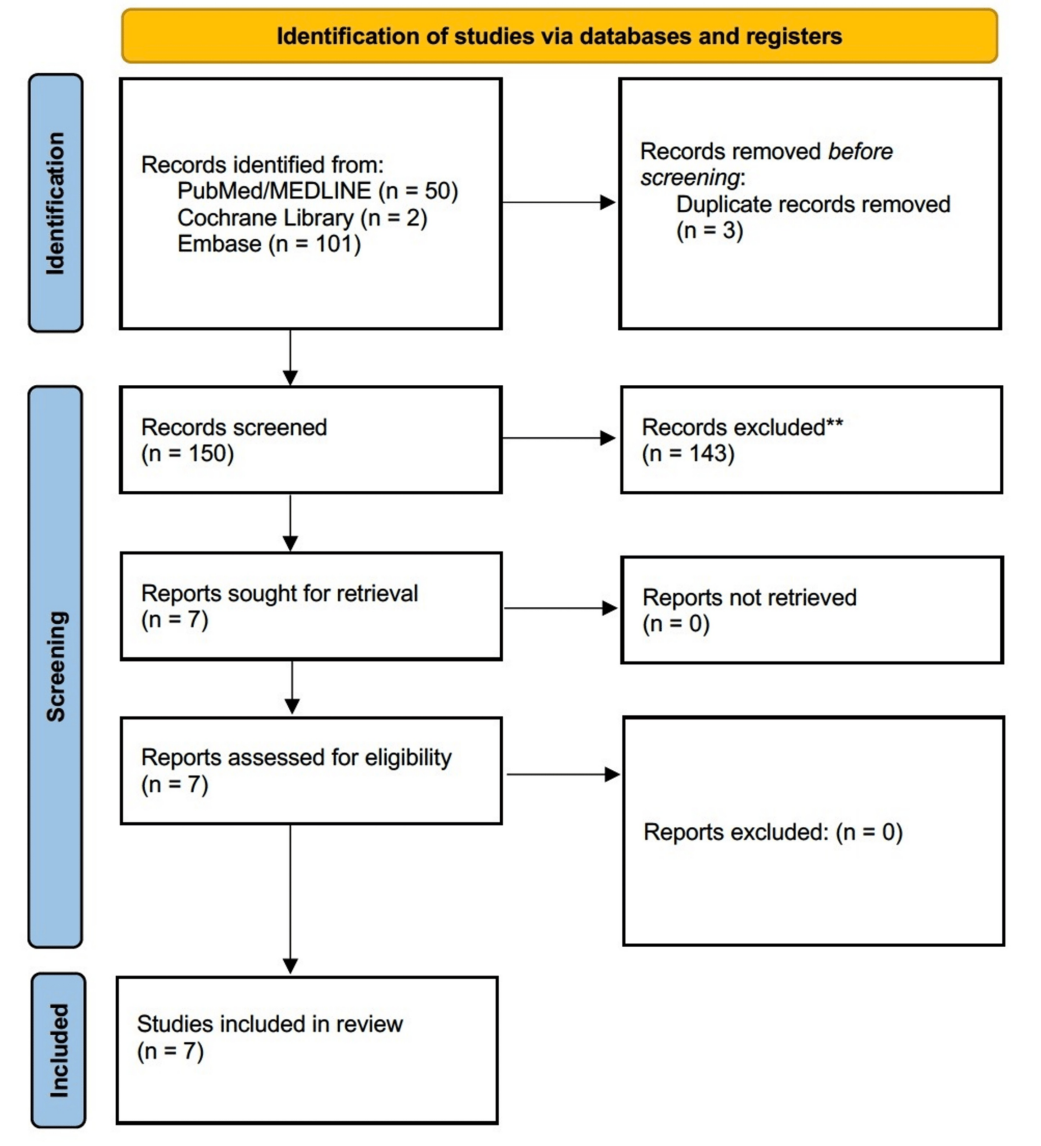
Preferred Reporting Items for Systematic Reviews and Meta-Analyses (PRISMA) 2020 flow diagram demonstrating study selection for the systematic review of internal herniation through the falciform ligament. A total of 150 records were identified through PubMed, Cochrane Library and Embase searching. Three duplicates were removed. After title and abstract screening, 143 records were excluded as per the criteria in the study eligibility section above. Seven full-text articles were assessed, and all met eligibility criteria, resulting in seven studies included in the qualitative synthesis. Exclusion criteria: Described diaphragmatic hernias (including Morgagni, Larrey or Bochdalek hernias) without true herniation through the falciform ligament. Reported ventral, incisional or epigastric hernias containing the falciform ligament rather than internal herniation. Focused on the surgical use of the falciform ligament as a flap, graft or buttress. Involved animal models or cadaveric/anatomical studies without clinical cases. Addressed other forms of internal hernia unrelated to the falciform ligament.

The database search identified multiple records, which were screened by title and abstract. After removal of duplicates and exclusion of clearly irrelevant articles, a subset of full-text articles was assessed for eligibility. Studies were excluded at the full-text stage if they described diaphragmatic hernias, ventral or incisional hernias involving the falciform ligament, use of the falciform ligament as a surgical flap or other types of internal hernia unrelated to the falciform ligament. Seven articles met the predefined inclusion criteria and were included in the qualitative synthesis. These consisted primarily of single-patient case reports and small imaging-focused case descriptions (Figure 1). 

Study Characteristics

All included studies were published within the preceding decade and written in English. The majority were case reports describing emergency presentations, while one article focused on characteristic CT findings in falciform ligament internal herniation. Reported cases originated from a range of geographic regions, reflecting the sporadic global reporting of this rare entity. No prospective cohort studies or comparative analyses were identified (Table 1). 

**Table 1:** Table of studies including background surgical history of patients, clinical presentation and imaging findings. F: female; M: male; SBO: small-bowel obstruction

Study	Year	Age/Sex	Prior Surgery	Presentation	Imaging
Vissers et al. [4]	2019	78 M	Laparoscopic; cholecystectomy	Severe abdominal pain	CT utilised
Matsuura et al. [6]	2023	90 M	None	Pain + vomiting; closed-loop SBO/strangulation suspected	CT: double beak + anterior-to-liver closed loop + caudal round ligament deviation
Raj Kumar et al. [7]	2021	75 M	None	Epigastric pain, distension, bilious vomiting	CT: loop of ileum through the parietal wall anterior to the liver.
Stephenson et al. [8]	2017	63 M	None	48 hours of epigastric pain	CT: anterior margin of liver, inflammatory fat concerning for necrosis
Baba et al. [9]	2021	15 M	None	Epigastric pain; worse supine, relieved with knee-chest	CT: round ligament/mesentery positional clue
Liang et al. [10]	2020	66 F	Open inguinal hernia + laparoscopic hysterectomy	Bulge in the upper abdomen, one year	Ultrasound: reported identified fat
Koussayer et al. [11]	2023	38 F	Prior umbilical hernia repair; myomectomy	Ventral bulge near the umbilicus	MRI: “incarcerated ventral hernia containing fat”

Patient Demographics

Patients ranged from adolescence to advanced age, with most reported cases occurring in older adults. Both sexes were represented. Several patients had no history of prior abdominal surgery, suggesting congenital defects of the falciform ligament, whereas others developed herniation through iatrogenic apertures following laparoscopic procedures, particularly cholecystectomy.

Clinical Presentation

Most patients presented acutely with symptoms consistent with small-bowel obstruction, including abdominal pain, nausea, vomiting and abdominal distension. In some cases, physical examination findings were nonspecific, contributing to diagnostic difficulty. A minority of patients reported posture-dependent pain, which prompted further radiologic evaluation and raised suspicion for internal herniation. Strangulation and bowel ischemia were described in several cases, emphasising the potentially life-threatening nature of delayed diagnosis. 

Imaging and Preoperative Diagnosis

CT was the principal diagnostic modality in all contemporary reports. Several studies highlighted characteristic CT features that aided preoperative suspicion, including a closed-loop segment of small bowel located anterior to the liver; beak-like tapering of bowel loops at the site of constriction; abnormal displacement of the round ligament; and mesenteric congestion or reduced bowel wall enhancement in strangulated cases. Despite these findings, definitive diagnosis was frequently established only at operative exploration (Table 2). 

**Table 2:** Table of studies including surgical findings, modality of surgery, bowel resection requirement and postoperative course.

Study	Intraoperative Finding	Approach	Bowel Resection	Repair/Defect Management	Outcome
Vissers et al. [4]	Small bowel herniation through an iatrogenic falciform defect	Laparoscopic exploration	Yes (necrosis → resection + anastomosis)	Recommend repair/division of the falciform if a defect created	Not detailed
Matsuura et al. [6]	Small bowel through falciform defect (L → R)	Laparotomy	No	Falciform dissected/opened to release strangulation	Preserved bowel; outcome not detailed
Raj Kumar et al. [7]	Small bowel through an falciform defect, gangrenous	Laparotomy	Yes	Falciform laid open	Uncomplicated
Stephenson et al. [8]	Infarcted fat	Laparoscopy	No	Repair without mesh	uncomplicated
Baba et al. [9]	Small bowel through the falciform ligament, viable	Laparoscopic	No	Falciform ligament cut to prevent recurrence	Not detailed in the abstract
Liang et al. [10]	Incarcerated fat	Laparoscopy	No	Repair with pre-peritoneal mesh	Uncomplicated, discharged on day 2 postoperative
Koussayer et al. [11]	Incarcerated falciform ligament + fat in ventral hernia	Robotic-assisted lap repair	No	Fascial closure + mesh	Discharged same day, uncomplicated

Operative Findings and Surgical Management

All included patients underwent emergency surgical intervention. Herniated contents consisted primarily of small bowel; in rare cases, greater omentum was reported. Surgical approaches varied and included both open laparotomy and laparoscopic exploration. Operative management typically involved reduction of the herniated viscus, followed by the division or closure of the falciform ligament defect to prevent recurrence. Bowel resection was required in patients with established ischemia or necrosis, whereas those with viable bowel underwent reduction alone. In iatrogenic cases, authors emphasised the importance of repairing or dividing falciform ligament defects created during laparoscopic procedures. 

Postoperative Outcomes

Short-term postoperative outcomes were generally favourable among reported cases. Patients without bowel necrosis typically recovered uneventfully after reduction and defect management. Those requiring intestinal resection also demonstrated good recovery following definitive surgery, including stoma reversal when performed. No long-term recurrence data were consistently reported, reflecting the limited follow-up inherent to isolated case reports. 

Discussion

This systematic review synthesises contemporary reports of internal herniation through defects in the falciform ligament, a distinctly rare cause of small-bowel obstruction that remains diagnostically challenging. The included studies, all published within the past decade, consist predominantly of single-patient case reports and imaging-focused descriptions, underscoring both the infrequency of the condition and the reliance on descriptive literature to guide clinical decision-making [3-8]. Despite the limited volume of data, several consistent themes emerged regarding patient presentation, imaging characteristics, operative management and outcomes. 

Clinical Presentation and Aetiology

Patients typically presented with acute abdominal pain, vomiting and features of small-bowel obstruction when the small bowel was found intraoperatively [4,7]. However, conversely, when fat or omentum was involved, presentation could be more subacute, with pain reported over prolonged periods [8]. Obstructive symptoms were nonspecific and frequently mimicked more common causes of obstruction, potentially contributing to delays in diagnosis. CT imaging in the acute setting was commonly reported and likely facilitated timely diagnosis and treatment when bowel integrity was compromised [6-8]. In several cases, strangulation and bowel ischemia were already present at the time of exploration, highlighting the potentially catastrophic consequences of missed or delayed recognition [4,7]. Both congenital and acquired defects of the falciform ligament were implicated. Congenital apertures may arise from incomplete fusion or resorption during embryologic development, whereas iatrogenic defects have been increasingly reported following laparoscopic surgery, particularly when trocars traverse or partially divide the falciform ligament [4,5]. The growing prevalence of minimally invasive hepatobiliary procedures may therefore contribute to a rising incidence of this entity, making awareness of falciform ligament internal herniation increasingly relevant to contemporary surgical practice [5].

Role of Computed Tomography 

Cross-sectional imaging, especially CT, played a central role in modern case detection. Although definitive diagnosis was still frequently made intraoperatively, several reports described reproducible radiologic patterns that may alert clinicians to this unusual diagnosis. These included closed-loop bowel segments positioned anterior to the liver, beak-like tapering at the point of constriction, abnormal displacement of the round ligament and signs of vascular compromise in strangulated loops [6]. Recognition of these features is important for both surgeons and radiologists, as early suspicion of an internal hernia through the falciform ligament may expedite operative management and reduce the risk of bowel necrosis. Systematic attention to the relationship between obstructed bowel loops and the falciform ligament on CT may therefore improve preoperative diagnostic accuracy [6,9].

Operative Strategies

All reported patients underwent surgical exploration, reflecting the inability to reliably manage this condition non-operatively once obstruction or strangulation is suspected. Open, laparoscopic and even robotic approaches were successfully employed, with minimally invasive surgery increasingly favoured in hemodynamically stable patients without advanced ischemia [4,6,10,11]. Operative management generally consisted of reduction of the herniated viscus followed by division or closure of the falciform ligament defect to prevent recurrence. When bowel viability was compromised, segmental resection was required, occasionally necessitating temporary stoma formation [4,7]. In contrast, patients with viable bowel typically recovered uneventfully after simple reduction and ligament division [6,10]. Although no comparative data exist to guide the choice of operative technique, the reviewed cases suggest that laparoscopy is feasible and safe in selected patients, provided there is a low threshold for conversion when ischemia or extensive distension is encountered [4,6,10].

Outcomes

Short-term postoperative outcomes were favourable across the included reports, even in patients requiring bowel resection [4,7]. Mortality was not reported in the screened contemporary cases, though historical series have described fatal outcomes, reinforcing the importance of timely surgical intervention [1,2]. Long-term follow-up and recurrence data were rarely provided, reflecting the inherent limitations of case-based literature [3-7].

Implications for Practice

Taken together, the available evidence supports a high index of suspicion for falciform ligament internal herniation in patients with unexplained proximal small-bowel obstruction, particularly when CT demonstrates an anterior closed-loop configuration adjacent to the liver or when prior laparoscopic instrumentation of the falciform ligament is documented [4-6]. It is, however, important to note that the majority of cases included had nil previous surgery in the upper abdomen or at all [6-11]. Surgeons performing upper-abdominal laparoscopic procedures should consider routine inspection and closure or division of inadvertent falciform ligament defects to reduce the risk of postoperative internal herniation [4,5]. Based on the synthesised cases, a pragmatic management approach would involve prompt operative exploration in symptomatic patients, careful assessment of bowel viability, division or closure of the falciform ligament defect and resection only when ischaemia is evident [4,6,7].

Study Limitations

This review is limited by the rarity of the condition and the exclusive reliance on case reports and small descriptive studies, which introduces publication bias and precludes quantitative synthesis. Heterogeneity in reporting limited consistent extraction of variables such as length of follow-up, recurrence rates and postoperative morbidity. Furthermore, restriction to English-language publications and a 10-year search window may have excluded additional relevant cases, although the aim of this review was to characterise contemporary practice. 

Future Directions

Given the ongoing expansion of laparoscopic surgery, further accumulation of cases in multicentre registries or collaborative databases may allow more robust characterisation of risk factors, diagnostic accuracy of imaging features and comparative outcomes between operative approaches. Standardised reporting of falciform ligament internal hernias, including long-term follow-up, would greatly enhance the evidence base for this rare but serious condition.

## Conclusions

Internal herniation through a defect in the falciform ligament is an exceptionally rare but potentially life-threatening cause of small-bowel obstruction. Contemporary reports over the past decade remain limited to isolated case descriptions, yet collectively highlight consistent patterns of presentation, radiologic features and operative management. Patients typically present with nonspecific symptoms, and delayed recognition may result in strangulation and bowel necrosis. CT plays a pivotal role in raising preoperative suspicion, particularly when closed-loop bowel is identified anterior to the liver in association with abnormal displacement of the round ligament. Once suspected, prompt surgical exploration is essential. Reduction of the herniated viscus with division or closure of the falciform ligament defect appears sufficient when bowel viability is preserved, whereas intestinal resection is required in cases of ischemia. Both open and minimally invasive approaches have been reported with favourable short-term outcomes.

Greater awareness of this rare entity among surgeons and radiologists may facilitate earlier diagnosis and intervention, especially in patients with prior upper-abdominal laparoscopic surgery. Further accumulation of cases through collaborative reporting and standardised documentation is necessary to better define diagnostic accuracy, optimal operative strategies and long-term outcomes for falciform ligament internal herniation.
